# The SAlzburg PEritoneal SUrface CAlculator (SAPESUCA): The First Web-Based Application for Peritoneal Surface Area Quantification

**DOI:** 10.3390/cancers15123134

**Published:** 2023-06-10

**Authors:** Tarkan Jäger, Philipp Schredl, Daniel Neureiter, Jaroslav Presl, Peter Tschann, Ingmar Königsrainer, Andreas Pascher, Klaus Emmanuel, Stephan Regenbogen, Jan Philipp Ramspott

**Affiliations:** 1 Department of Surgery, Paracelsus Medical University Salzburg, 5020 Salzburg, Austria; 2 Institute of Pathology, Paracelsus Medical University Salzburg, 5020 Salzburg, Austria; 3 Department of General and Thoracic Surgery, Academic Teaching Hospital Feldkirch, 6800 Feldkirch, Austria; 4 Department for General, Visceral and Transplant Surgery, University Hospital Muenster, 48149 Muenster, Germany; 5 Department for Trauma Surgery, BG Trauma Center Murnau, 82418 Murnau, Germany; 6 Department for Trauma Surgery, BG Trauma Center Ludwigshafen, University of Heidelberg, 67071 Ludwigshafen am Rhein, Germany

**Keywords:** peritoneal metastases, cytoreductive surgery, hyperthermic intraperitoneal chemotherapy, peritoneal surface area, intraperitoneal drug dosing, peritoneal cancer index, peritoneal surface malignancies, R Shiny app, web-based application

## Abstract

**Simple Summary:**

The combination of cytoreductive surgery and hyperthermic intraperitoneal chemotherapy (HIPEC) is considered a promising therapeutic strategy in selected patients with peritoneal metastases. The recent randomized phase III PRODIGE 7 trial did not show any survival benefit of adding 30-min oxaliplatin-based HIPEC to cytoreductive surgery in colorectal cancer patients with peritoneal metastases and has triggered the discussion of HIPEC application. Cytoreductive surgery goes ahead with high inter- and intra-individual resection variabilities of the affected organs and their associated peritoneal surface areas. The diverse range of HIPEC protocols with differing dosing regimens complicates uniform implementation. Moreover, a quantification of the resected and remaining peritoneal surface area during cytoreductive surgery is lacking so far. In this article, we present the first web-based app for standardized and detailed documentation of peritonectomy extent and determination of the peritoneal surface-dependent HIPEC dose: The SAlzburg PEritoneal SUrface CAlculator (SAPESUCA). SAPESUCA is one valuable contribution towards the urgently needed standardization of cytoreductive surgery and HIPEC.

**Abstract:**

(1) Background: Peritoneal metastasized colorectal cancer is associated with a worse prognosis. The combination of cytoreductive surgery (CRS) and hyperthermic intraperitoneal chemotherapy (HIPEC) showed promising results in selected patients, but standardization is lacking so far. We present the first tool enabling standardized peritoneal surface area (PSA) quantification in patients undergoing CRS and HIPEC: The SAlzburg PEritoneal SUrface CAlculator (SAPESUCA). (2) Methods: SAPESUCA was programmed using the R-Shiny framework. The application was validated in 23 consecutive colon cancer patients who received 27 closed oxaliplatin-based HIPECs between 2016 and 2020. The programming algorithm incorporates the patient’s body surface area and its correlated peritoneal surface area (PSA) based on the 13 Peritoneal Cancer Index (PCI) regions. (3) Results: Patients’ median age was 56 years. Median PCI was 9. SAPESUCA revealed a mean PSA of 18,613 cm^2^ ± 1951 of all patients before compared to 13,681 cm^2^ ± 2866 after CRS. The Central PCI region revealed the highest mean peritonectomy extent (1517 cm^2^ ± 737). The peritonectomy extent correlated significantly with PCI score and postoperative morbidity. The simulated mean oxaliplatin dose differed significantly before and after CRS (558 mg/m^2^ ± 58.4 vs. 409 mg/m^2^ ± 86.1; *p* < 0.0001). (4) Conclusion: SAPESUCA is the first free web-based app for standardized determination of the resected and remaining PSA after CRS. The tool enables chemotherapeutic dose adjustment to the remaining PSA.

## 1. Introduction

Peritoneal metastases of colorectal cancer appear in 4–8% of patients at diagnosis (synchronous), whereas 20% develop metachronous peritoneal metastases during follow-up appointments [1,2]. The presence of peritoneal metastases is associated with reduced overall survival (OS) (16.3 months) compared with non-peritoneal metastases (19.1 months for liver metastases) [3,4]. Cytoreductive surgery (CRS) combined with hyperthermic intraperitoneal chemotherapy (HIPEC) is a promising therapeutic option but has triggered a wide discussion due to actual study results [5,6,7]. The most recent PRODIGE 7 trial did not show any survival benefit in adding 30-min oxaliplatin-based HIPEC (closed (360 mg/m^2^) or open (460 mg/m^2^) abdomen techniques), combined with systemic chemotherapy (400 mg/m^2^ fluorouracil and 20 mg/m^2^ folinic acid) to CRS in patients with colorectal peritoneal metastases compared with CRS only (41.7 months vs. 41.2 months). Moreover, patients receiving HIPEC showed more postoperative late complications. Therefore, it was assumed that CRS alone, with the aim of maximal cytoreduction, should be the therapeutic cornerstone in this patient group [7].

One of the main challenges of CRS remains the high inter- and intra-individual resection variability of the peritoneal surface area (PSA). Moreover, a lack of HIPEC standardization (if added to CRS) due to different therapeutic protocols based on several parameters, such as choice of the chemotherapeutic agent, dosing regimen, procedure duration, or delivery technique [8,9,10], may lead to varying study results and controversial benefits.

To determine the extent of peritoneal disease, whether a surgical approach is needed, and its extent and prognosis in the case of peritoneal metastases, different classification and scoring systems are described. In colorectal cancer, the Peritoneal Cancer Index (PCI) Score [11], the Simplified Peritoneal Cancer Index (SPCI) Score [12], and the Gilly Score [13] are the main scores to determine peritoneal disease extent. In contrast, the Completeness of Cytoreduction (CC) Score [11] and R-Score [14,15,16] describe the achieved cytoreduction. The Prior Surgical Score (PSS) [11] and Younan Score [17] were developed to characterize the extent of surgical procedures.

In 1996, Jacquet and Sugarbaker first described the PCI score, which describes the number, size, and distribution of peritoneal carcinomatosis throughout 13 abdominopelvic regions. Peritoneal lesion size is defined as follows: LS 0: No tumor seen, LS 1: Tumor up to 0.5 cm, LS2: Tumor up to 5.0 cm, LS3: Tumor > 5.0 cm or confluence. It is the most precise, validated, and applied score [11]. In colon cancer patients with a PCI score greater than 20, palliation is defined as the treatment goal [18]. In the modified PCI score, the SPCI Score, 13 PCI regions are reduced to only seven anatomical regions [12]. Another tool, the Gilly carcinomatosis staging, combines the peritoneal cancer size and its distribution. The five stages range from no macroscopic disease (stage 0) to large malignant nodules (stage 4) [13].

To describe the achieved cytoreduction in patients with peritoneal carcinomatosis after surgery, the Completeness of Cytoreduction (CC) score incorporating three categories with complete (CC-0: No visible disease or CC-1: Tumor nodules < 0.25 cm) or incomplete (CC-2: Tumor residual ≥ 0.25 ≤ 2.5 cm or CC-3: Tumor residual > 2.5 cm) cytoreduction was developed [11]. In contrast to the international standardized CC-Score, the R-Score is defined differently and, therefore, is less applied [14,15,16].

Two more scores are available to characterize the extent of previous surgical procedures before cytoreduction in colorectal cancer patients. The Prior Surgical Score (PSS) incorporates nine of thirteen abdominopelvic PCI regions and the number of dissected regions (PSS-0–PSS-3). Here, PSS-0 means that no prior surgery or only a biopsy was performed, PSS-1 describes one region with prior surgery, PSS-2 indicates two to five regions, and PSS-3 indicates more than five regions previously dissected [11]. In the extent of surgery score (Younan Score), cytoreduction was classified into three levels (I-III). The higher the level, the more cytoreductive procedures were performed [17].

All scores aim to select patients for adequate cancer therapy, determine their prognosis, and consider different parameters. So far, no score fully describes the total affected PSA before and the resected PSA after CRS. Therefore, we developed the first web-based application for peritoneal surface quantification in CRS and HIPEC, including aspects of each established peritoneal metastasis classification system: The SAlzburg PEritoneal SUrface CAlculator (SAPESUCA). The aim of this Shiny app is to quantify and visualize patients’ PSA before and after CRS. The app provides interactive plots, summary tables, and final reports. Furthermore, we technically now have the possibility to adjust the HIPEC dose to the remaining PSA.

## 2. Materials and Methods

### 2.1. SAPESUCA R Shiny Application

**Figure 1:**
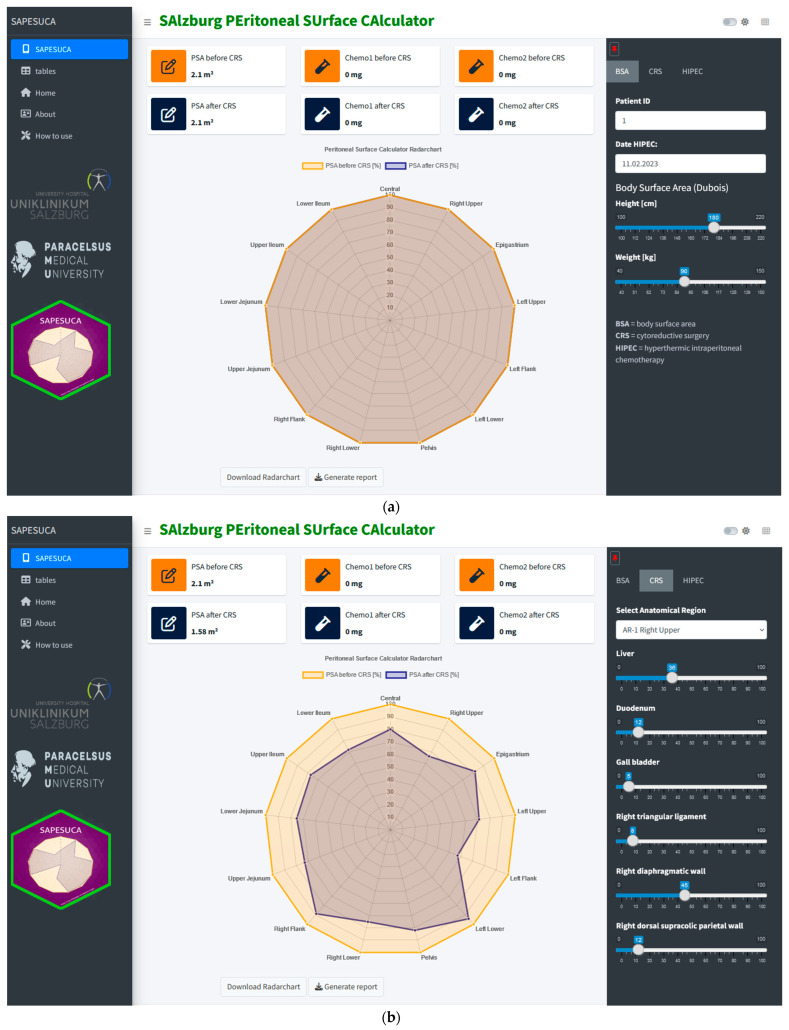

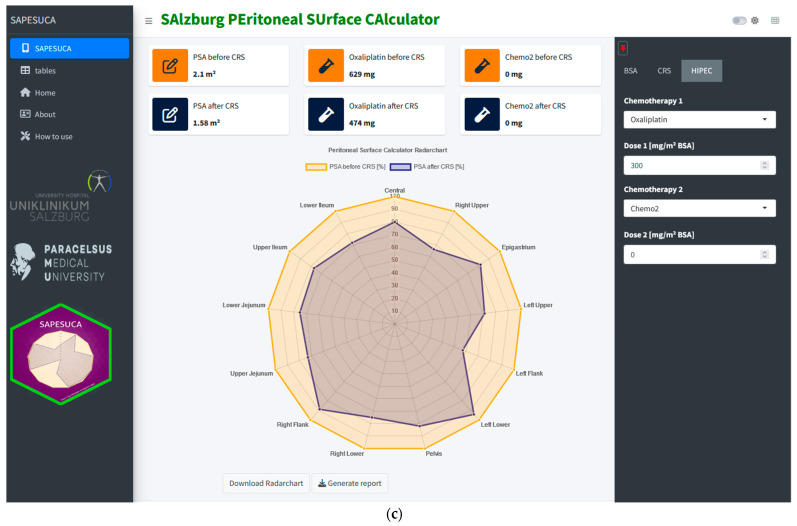
(**a**) Screenshot of the app’s landing page. The user has the option to pin the right sidebar. On the body surface area (‘BSA’) tap, patient’s ID, date of hyperthermic intraperitoneal chemotherapy (HIPEC), and patient’s height (cm) and weight (kg) data are inserted. (**b**) Screenshot of the app’s graphical user interface (GUI). On the cytoreductive surgery (‘CRS’) tap, the user has the option to access all 13 Peritoneal Cancer Index (PCI) regions within the drop-down menu. The corresponding anatomical surface areas of the chosen PCI region are shown as slider input fields. Manipulation of the slider inputs defines the amount of resection in percent (range 0% to 100%). Key Performance Indicators (KPI) such as peritoneal surface area (PSA) before and after CRS (in cm^2^) are calculated automatically. Data are also shown simultaneously in a radar chart. (**c**) Screenshot of the app’s graphical user interface (GUI). On the hyperthermic intraperitoneal chemotherapy (‘HIPEC’) tap, the user must choose the chemotherapeutic drug and insert its dose (mg/m^2^). Key Performance Indicators (KPI) such as HIPEC drug doses (in mg) before and after cytoreduction are calculated automatically.

SAPESUCA was built using R version 4.2.2 [2022-10-31 ucrt] [19] and the Shiny library version 1.6.0. Other libraries used include golem, bs4Dash, shinythemes, radarchart, shinyscreenshot, rmarkdown, markdown, fontawesome, pkgload, pkgdown2.0.7, shinyjs, and grDevicesdplyr. The user interface of the application is designed to be intuitive and user-friendly, with a main dashboard that provides an overview of the calculated data combined with a left and right sidebar (Figure 1). The calculated values are presented with one dynamic radar chart and six dynamic Key Performance Indicator (KPI) summary widgets. The interface also includes interactive tables, as well as options to download data and results from reports. The app was deployed and hosted using the shinyapps.io platform [20]. The server was configured to allow access to the app from anywhere with an internet connection (https://taja.shinyapps.io/sapesuca-app/, version 0.0.0.9000, accessed on 31 January 2023). For full details, instructions, and examples, refer to the package site (https://tarjae.github.io/sapesuca/ (accessed on 31 January 2023)).

### 2.2. SAPESUCA Calculation Algorithm

The programming algorithm is a modification of the initial software solution PEritoneal SUrface CAlculator (PESUCA), which was recently described in detail [21]. It incorporates the patient’s body surface area (BSA), its correlated PSA, and the calculation of the relative proportion of different anatomical regions to the total PSA before CRS.

SAPESUCA is a simplification of PESUCA based on 13 peritoneal regions in analogy to the PCI score [11]. To achieve this simplification, the initial calculation algorithm which is based on 40 anatomical regions defined by Albanese et al. [22], was inherited into the 13 peritoneal regions defined by Jacquet and Sugarbaker [11]. Two of the forty anatomical regions (Mesentery and Jejunum-ileum) were each split into Mesentery Upper/Lower Jejunum and Mesentery Upper/Lower Ileum, and Upper/Lower Jejunum and Upper/Lower Ileum. These regions were incorporated into the following PCI regions; 9: Upper Jejunum, 10: Lower Jejunum, 11: Upper Ileum, and 12: Lower Ileum. Furthermore, the anatomical regions right and left of the anterolateral infraumbilical and supraumbilical wall were incorporated into the PCI region 0: Central (Table S1). The body surface area is calculated using the DuBois formula: Surface (m^2^) = 0.20247 × height (m)^0.725^ × weight (kg)^0.425^ [23].

All regrouped regions were included in the app as drop-down menu items and slider input fields in the dynamic right sidebar, which only shows up when the main dashboard (SAPESUCA) is active.

On the landing page, the user has the option to pin the right sidebar. On the ‘BSA’ tap, the patient’s ID, date of HIPEC, patient’s height (cm) and weight (kg) are inserted (Figure 1a). The next step is to open the ‘CRS’ tab on the right sidebar. The 13 PCI regions can be accessed within the drop-down menu. After selecting one region, the corresponding anatomical surface areas are shown as slider input fields. By manipulating the slider inputs, the user can define the amount of resection in percent (range 0% to 100%). Simultaneously, the software algorithm calculates the KPI, such as PSA before and after CRS (in cm^2^). Moreover, the app automatically shows these data in the dynamic radar chart (Figure 1b).

By selecting the ‘HIPEC’ tap, the user must choose the chemotherapeutic drug (two drugs can be chosen simultaneously) from the drop-down menu and must insert its dose (mg/m^2^). SAPESUCA then automatically calculates the PSA-based HIPEC drug doses before and after cytoreduction (in mg) (Figure 1c).

Furthermore, the app gives the option to export and save detailed plots and csv files of the calculated areas. Via the ‘Download Radarchart’ and ‘Generate report’ buttons, a report is generated.

SAPESUCA equates BSA with PSA if no values are inserted in any of the anatomical regions. This is in accordance with Albanese et al., who have shown and broadly discussed that the PSA can be estimated from BSA formulas [22].

In this section, only the main features of the app are discussed; however, a detailed user manual can be found on the package site (https://tarjae.github.io/sapesuca/, accessed on 31 January 2023).

### 2.3. SAPESUCA Simulation Validation

The app was tested using the data of 23 patients with peritoneal metastasized colon cancer who underwent 27 CRS and closed HIPEC procedures between 2016 and 2020 to ensure that the results were accurate and reliable. The testing procedures included both manual testing by the development team and automated testing using the Shinytest library in R. The results of the tests showed that the app produces consistent and accurate results, as presented in the results and discussed in the discussion section.

### 2.4. Patients’ Selection Criteria

Out of 160 CRS and HIPEC procedures between 2012 and 2022, 23 consecutive colon cancer patients with histologically proven peritoneal metastases who underwent 27 CRS and closed HIPEC procedures between 2016 and 2020 were selected for software simulation and validation.

All patients underwent a thorough clinical examination, blood tests, esophagogastroduodenoscopy and colonoscopy, diagnostic laparoscopy (in cases with uncertain peritoneal disease extent), and a computed tomography (CT) scan before surgery. More than three liver metastasis, infiltration of the small bowel (>60% affected), mesenteric axis, pancreatic head or retroperitoneal plane, and PCI > 21 were considered irresectable.

Clinicopathological information was obtained from our prospectively managed HIPEC database. Perioperative complications were classified according to the Clavien-Dindo Classification [24]. The study was performed in accordance with the Helsinki Declaration of 1975, as revised in 1983 and according to the guidelines of the local institutional board and the ethics committee. All patients signed an informed consent at the time of admission.

### 2.5. Cytoreductive Surgery

On admission, patients’ baseline characteristics were directly integrated into our prospectively maintained HIPEC registry. Furthermore, patients’ height and weight data were integrated into SAPESUCA to determine patients’ BSA and their correlated PSA.

CRS was performed by experienced senior surgical oncologists with resection of all tumor-infiltrated structures along with peritonectomy procedures after mid-line laparotomy from the xiphoid process to the symphysis pubis in our tertiary peritoneal cancer care center. The aim was complete cytoreduction and describe in analogy to the Completeness of Cytoreduction score (CC-0: No visible disease, CC-1: Tumor nodules < 0.25 cm, CC-2: Tumor residual ≥ 0.25 ≤ 2.5 cm, or CC-3: Tumor residual > 2.5 cm) [11]. Liver metastases were ruled out by digital examination. Intraoperative ultrasound examination was performed in any case of suspicion.

During CRS, an assistant in the operating room instantaneously entered the amount of each resected peritoneal area (in %) in SAPESUCA. If no peritoneal resection was performed, 0% was entered, whereas 100% is defined as maximal cytoreduction in the corresponding anatomical region. Regions in which previous surgical cancer procedures were performed were classified accordingly. Thus, higher percental numbers represent a greater peritonectomy extent. In parallel, the PCI score was determined.

In cases of unresectable tumor spread due to anatomical location, including mesenteries of the small and the large bowel, lesions were destroyed with a high-voltage monopolar device. After CRS, SAPESUCA automatically determined the residual PSA and the associated peritonectomy extent in total and in the different PCI regions. Furthermore, SAPESUCA automatically calculated the PSA-based intraperitoneal chemotherapeutic drug dose.

### 2.6. HIPEC Procedure

After complete cytoreduction, closed HIPEC with oxaliplatin 300 mg/m^2^ was administered for 30 min at 41 °C. Simultaneously intravenous 5-fluorouracil (400 mg/^2^) and folinic acid (20 mg/m^2^) were administered to maximize the effect of oxaliplatin as described by Elias et al. [25].

### 2.7. Postoperative Systemic Therapy and Follow-Up

Depending on the patient’s individual medical history and the consensus of the interdisciplinary tumor board, the patient received postoperative systemic chemotherapy. Follow-up was carried out every three months for the first two years and, after that, annually. We performed a chest and/or abdominal CT scan for any sign of recurrence.

### 2.8. Statistical Analysis

Data were analyzed using R (version 4.2.2) software [19]. Descriptive statistics were computed to summarize the clinicopathological characteristics. Independent sample t-tests or Wilcoxon rank-sum test were used to compare the mean scores between the PSA and chemotherapeutic drug dose before and after CRS. Pearson’s correlation coefficient was calculated to examine the relationship between two continuous variables. Normality assumption was checked using the Shapiro-Wilk test and visual inspection of the residuals. The assumption of homogeneity of variances was tested using Levene’s test. All tests were performed at a significance level of 0.05, and results were considered statistically significant if *p* < 0.05.

## 3. Results

### 3.1. Patients’ Baseline Characteristics

In total, 23 patients with peritoneal metastasized colon cancer who underwent 27 CRS and HIPEC procedures between 2016 and 2020 were included for software validation. Patients’ baseline characteristics are presented in detail in Table 1. Patients’ median age was 56 years (range 22–75 years). More than half of the patients were male (*n* = 16, 59%). Four patients underwent two CRS and HIPEC procedures due to tumor relapse. Histologically, twelve specimens (44%) showed adenocarcinoma and mucinous differentiation.

In most patients, the tumor was localized in the sigmoid colon (*n* = 10, 37%), followed by the right colon (*n* = 8, 30%). The median PCI score was 9 (range 1–21). Before CRS, 67% of the patients (*n* = 18) had undergone previous abdominal surgery for cancer and had received previous chemotherapy. The median operative time was 352 min (range: 190–632 min). Optimal CRS with CC 0 could be achieved in 26 patients (96%). One patient revealed CC 1 after surgery. The most resected organs were colon (*n* = 21, 17%) and the greater omentum (*n* = 20, 16%). Two pancreatectomies (4%) and five splenectomies (19%) were performed. No patient underwent gastrectomy, and in only one patient (4%), cystectomy was necessary. A stoma was created in half of the patients (*n* = 14). After CRS, all patients underwent closed 41 °C-HIPEC with oxaliplatin 300 mg/m^2^ over 30 min bidirectional with intravenous 5-fluorouracil 400 mg/m^2^ and 20 mg/m^2^ BSA folinic acid. The median hospital stay was 14 days (range: 7–61 days). In 27 CRS and HIPEC procedures, the 30-day complication rate was 37% (*n* = 10) with 15% (*n* = 4) grade IIIb complications according to the Clavien-Dindo Classification (Table 1).

### 3.2. Quantification of the Peritoneal Surface Area before and after Cytoreductive Surgery with the SAPESUCA Application

To simulate and validate SAPESUCA, we extracted the data from 27 consecutive CRS and HIPEC procedures of peritoneal metastasized colon cancer patients between 2016 and 2020. All patients received closed oxaliplatin-based HIPEC after CRS.

**Figure 2:**
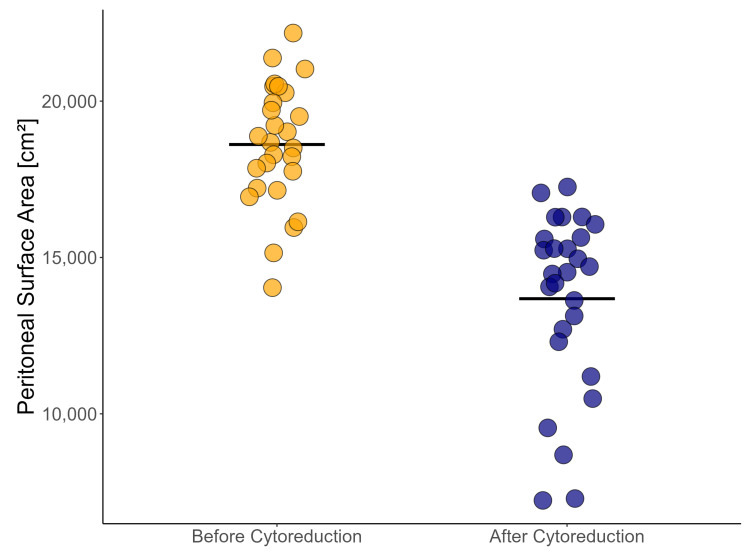
Peritoneal surface area (cm^2^) before (orange) and after (navy) cytoreduction in 27 cytoreductive surgery procedures. Mean is shown.

SAPESUCA quantified the resected and remaining PSA after CRS. The overall mean PSA was 18,613 cm^2^ ± 1951 before CRS and 13,681 cm^2^ ± 2866 after CRS (Figure 2).

As SAPESUCA is able to quantify the peritonectomy extent in all 13 peritoneal regions in analogy to the PCI score [11], a detailed analysis and quantification of each PSA of these regions before and after CRS was performed.

**Figure 3:**
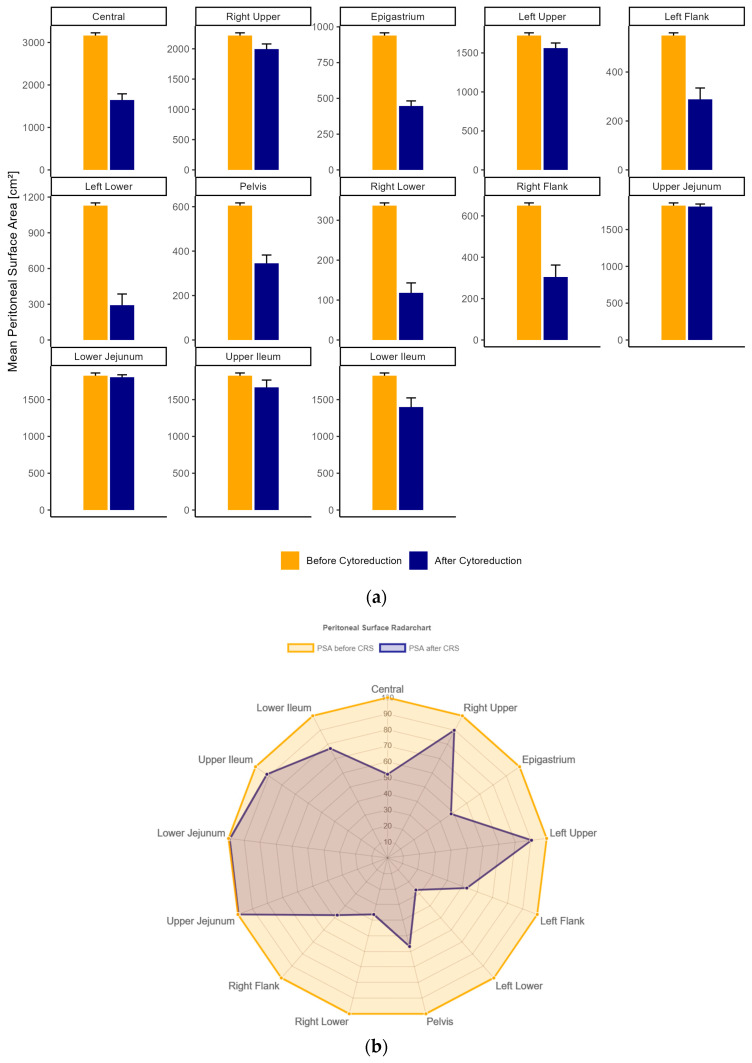
(**a**) Total mean peritoneal surface area (cm^2^) before (orange) and after (navy) cytoreductive surgery in all Peritoneal Cancer Index (PCI) regions. Data of all procedures are shown as mean ± SD. (**b**) Radar chart of all procedures with mean percental resected peritoneal surface areas (PSA) in all PCI regions. Descending numbers (100% to 0%) represent the peritonectomy extent in %. Orange color indicates PSA before, whereas navy color indicates PSA after cytoreductive surgery.

The Central PCI region (mean PSA 3163 cm^2^ ± 332), followed by the Right Upper region (2219 cm^2^ ± 232), and Upper/Lower Jejunum/Ileum (1825 cm^2^ ± 191) revealed the largest PSA before CRS. After CRS, the Right Upper region (1995 cm^2^ ± 440), the Upper (1812 cm^2^ ± 179) and Lower Jejunum (1804 cm^2^ ± 171) had the largest mean PSA. The right lower (337 cm^2^ ± 35), Left Flank (549 cm^2^ ± 57), and Pelvis (605 cm^2^ ± 64) had the lowest mean PSA before CRS. After CRS, the lowest PSA was detected in the Right Lower (118 cm^2^ ± 129), Left Flank (288 cm^2^ ± 240), and Left Lower region (292 cm^2^ ± 487) (Figure 3a, Table S2). Mean percental reduction of all PSAs in each PCI region after CRS calculated and graphically outputted by SAPESUCA is shown in Figure 3b.

### 3.3. Quantification of the Peritonectomy Extent with the SAPESUCA Application

**Figure 4:**
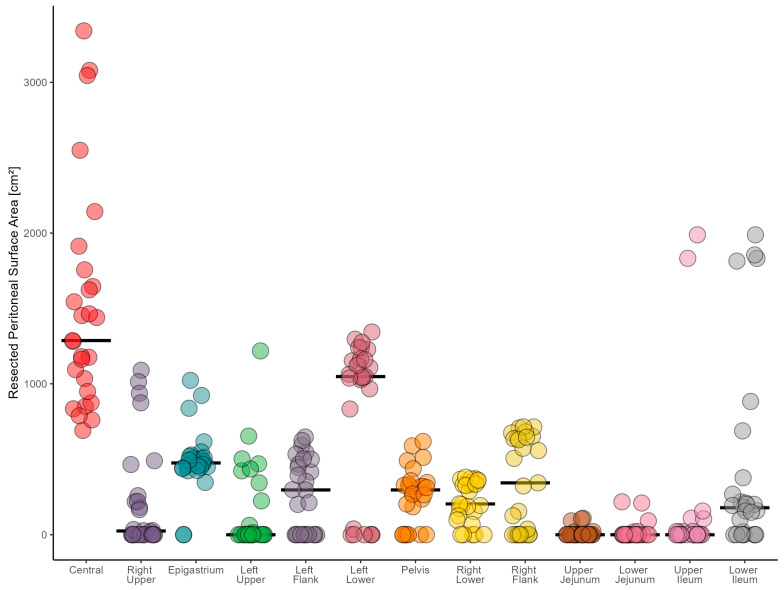
Resected peritoneal surface area (cm^2^) of each Peritoneal Cancer Index (PCI) region. Each dot represents one cytoreductive surgery procedure. Mean is shown.

The greatest peritonectomy extent was found in the Central PCI region with a mean resected peritoneal surface area of 1517 cm^2^ ± 737. The Left Lower PCI region showed the second largest extent with 836 cm^2^ ± 512. The third highest peritonectomy extent was revealed in the Epigastrium PCI region with 492 cm^2^ ± 209. The upper jejunum had the lowest peritonectomy extent (13 cm^2^ ± 32) (Figure 4, Table S3).

### 3.4. Individual Quantification of the Peritoneal Surface Area before and after CRS

**Figure 5:**
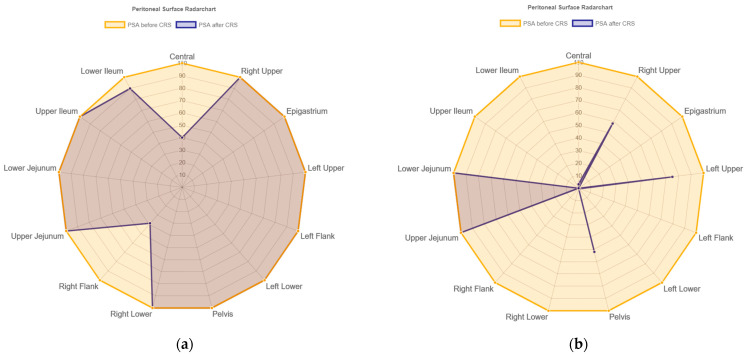
(**a**,**b**) Radar charts of two individual patients’ resected peritoneal surface areas in all Peritoneal Cancer Index (PCI) regions calculated and graphically outputted by SAPESUCA. Descending numbers (100% to 0%) represent the peritonectomy extent in %. Orange color indicates PSA before, whereas navy color indicates PSA after cytoreduction. (**a**) Radar chart of a 74-year-old female patient (PCI score: 9) with less extensive peritonectomy. (**b**) Radar chart of a 61-year-old male patient (PCI score: 19) with more extensive peritonectomy.

Two individual patients with peritoneal metastasized transverse colon cancer were selected to show the large resection variability in the study cohort determined by SAPESUCA (Figure 5). The first patient is a 74-year-old female with adenocarcinoma and a PCI score of 9. The PSA before CRS was 15,146 cm^2^. The PSA decreased to 13,129 cm^2^ after CRS, which means a percental reduction of 13% (2017 cm^2^). The Central and Right Flank region revealed the largest peritonectomy extent (Figure 5a). Another 61-year-old male patient with adenocarcinoma and a PCI score of 19 showed a strong decrease of the PSA due to large peritonectomy extent from 20,272 cm^2^ before to 7234 cm^2^ after CRS, which means a percental reduction of 64% (13,038 cm^2^). The regions of the upper and lower jejunum showed the lowest peritonectomy extent (Figure 5b).

### 3.5. Correlation of Peritonectomy Extent and PCI Score

**Figure 6:**
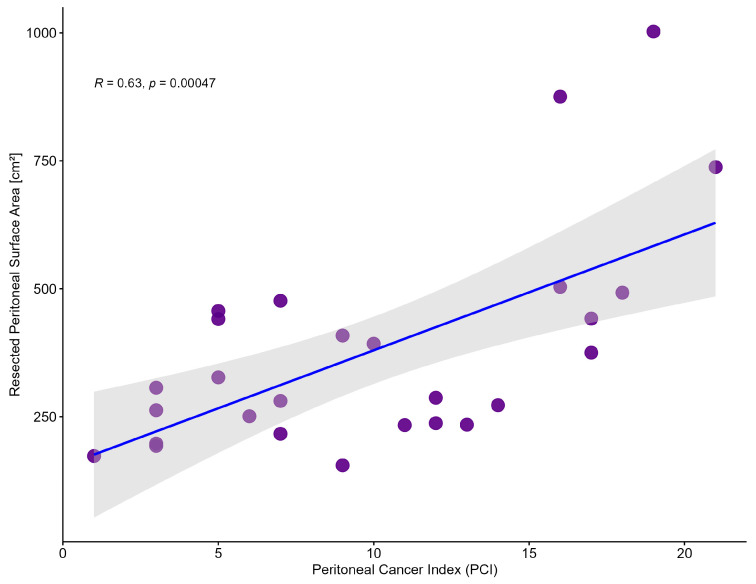
Correlation of Peritoneal Cancer Index (PCI) values and resected peritoneal surface areas [cm^2^]. Grey shadow shows 95% confidence interval. Each dot represents one cytoreductive surgery procedure (Pearson correlation).

As SAPESUCA is based on the 13 PCI regions, we determined the correlation between the PCI values of all patients and their peritonectomy extent. The quantification highlighted a direct correlation between the peritonectomy extent and PCI score (Pearson R = 0.63, *p* = 0.00047) (Figure 6).

### 3.6. Correlation of Peritonectomy Extent and Morbidity

**Figure 7:**
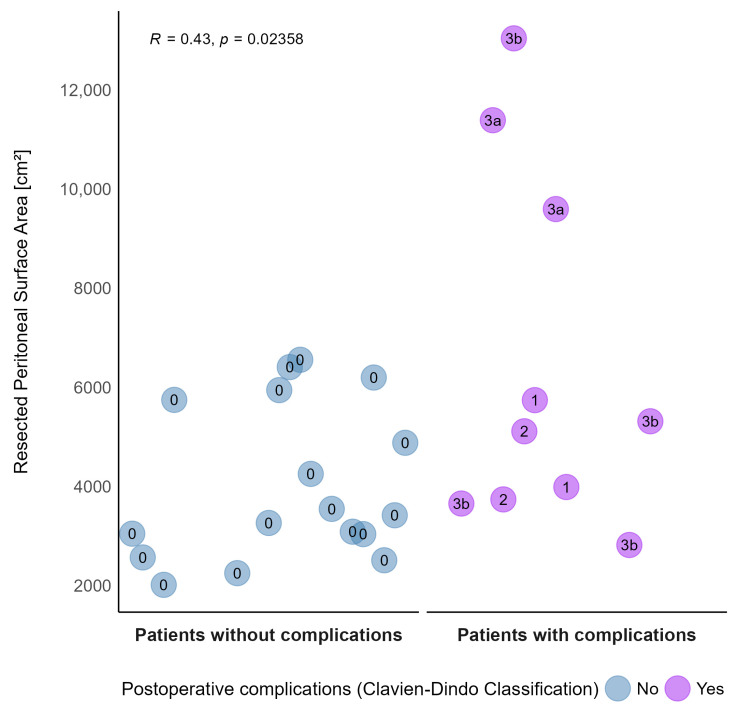
Correlation of Clavien-Dindo postoperative complication grades (0-3b) and resected peritoneal surface areas [cm^2^]. Blue color indicates no complication (grade 0), whereas purple color indicates a complication (grade 1-3b). Each dot represents one cytoreductive surgery procedure (Pearson correlation).

As the extent of cytoreduction is a well-known risk factor for postoperative morbidity, we performed a point-biserial correlation of peritonectomy extent calculated by SAPESUCA and morbidity according to the Clavien-Dindo Classification [24]. Our analysis revealed a direct correlation between the peritonectomy extent and postoperative morbidity with a point-biserial correlation coefficient of 0.43 (*p* = 0.02358) (Figure 7).

### 3.7. Simulation of the Chemotherapeutic Dose Adjustment by SAPESUCA Based on the Remaining PSA after CRS

**Figure 8:**
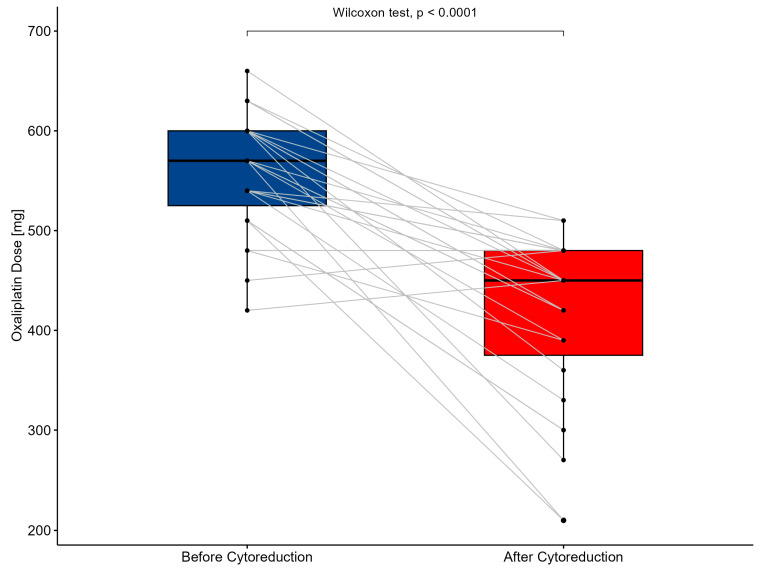
Boxplot of oxaliplatin-based HIPEC dose before (blue) and after cytoreductive surgery (red) calculated by SAPESUCA. Data of all procedures are shown.

SAPESUCA was programmed to determine and simulate the chemotherapeutic HIPEC dose based on the patient’s PSA before (equivalent to BSA as shown by Albanese et al. [22]) and after CRS. The mean oxaliplatin dose in all 23 colon cancer patients before CRS (mean 558 mg/m^2^ ± 58.4) differed significantly from the simulated dose after CRS (mean 409 mg/m^2^ ± 86.1) (*p* < 0.0001) (Figure 8).

## 4. Discussion

The development of the combined CRS and HIPEC treatment strategy has innovated the surgical treatment of peritoneal surface malignancies. Whereas this therapeutic approach represents the state-of-the-art therapy for peritoneal mesothelioma and pseudomyxoma peritonei [26,27,28,29], HIPEC in colorectal peritoneal metastases is discussed controversially due to recent study results, mainly the PRODIGE 7 trial [7].

As a result of individual peritoneal tumor spread, surgeons are confronted with a high inter- and intra-individual resection variability of the PSA during CRS. Several peritoneal carcinomatosis staging systems are available. Among these, the most applied PCI score precisely determines carcinomatosis implants within the abdominopelvic cavity, whereas the four-tier CC-Score describes the achieved cytoreduction [11]. So far, a sole standardized tool predicting both the peritoneal disease and the following peritonectomy extent is lacking. Here, we present such a tool. SAPESUCA is the first web-based application enabling individual PSA quantification before and after CRS based on the PCI score [11].

Here, we have shown the feasibility of detailed PSA quantification before and after surgery and revealed a direct correlation between the PCI score and the peritonectomy extent. In our cohort, the occurrence of postoperative complications correlated positively with the extent of peritoneal surface resection. This is in line with multiple studies that have shown a predictive value for postoperative complications of variables correlated with CRS extent as the number of peritonectomy procedures [30,31,32,33,34].

The direct correlation between the PCI score and peritonectomy extent shown here means to accept the PCI’s disadvantages. On the one hand, a lower PCI and, thus, a lower peritonectomy extent means lower tumor burden and, therefore, probably a more favorable prognosis. On the other hand, lower peritonectomy extent could mean invasive cancer at crucial anatomic sites with impossible complete cytoreduction and, therefore, a more unfavorable situation. Neither the PCI score nor the SAPESUCA tool are fully capable of describing situations adequately, like what was mentioned above. Moreover, the major requirements of a quantitative prognostic indicator such as SAPESUCA are simplicity and reproducibility within a defined population of patients. The exclusion of peritonectomy estimation bias still needs to be shown in ongoing research and external validation.

As clinical staging, mainly based on imaging, still does not adequately determine actual peritoneal tumor burden, precise intraoperative staging tools are extremely important. SAPESUCA quantifies the cytoreduction and might therefore be a valuable prognostic tool as the prognosis of peritoneal surface malignancies mainly depends on the complete cytoreduction. Further studies are needed to determine if SAPESUCA is a similar prognosis indicator and precise staging tool compared with the PCI score. The recent PRODIGE 7 trial failed to show any survival benefit in colorectal cancer patients with peritoneal metastasis undergoing 30-min oxaliplatin-based HIPEC [7]. The study was criticized due to methodological weaknesses such as an underestimation of the cytoreductive surgery effect alone, uncertain oxaliplatin efficacy, and possible adverse effects of the carrier solution and hyperthermia [35]. PRODIGE 7 is one more study reflecting the actual challenges in HIPEC therapy, as too many parameters are still under debate affecting HIPEC efficacy [8,9,10]. Here we suggest one more possible explanation for this lack of benefit. One of the most controversial issues is the choice of chemotherapeutic dosing regimen: BSA-based protocols with fixed chemotherapeutic doses (mg/m^2^) diluted in different carrier solution volumes or concentration-based protocols with chemotherapeutic drug doses and carrier solution volumes based on BSA. Based on the Dedrick model [36], we hypothesize that less intraperitoneal chemotherapeutic drug is eliminated from the peritoneal cavity after extensive peritoneal resection due to a decrease in the peritoneal contact area and therefore leads to higher intraperitoneal concentrations associated with more postoperative complications. Currently, HIPEC dosing is based on the patient’s BSA and does not consider the patient’s PSA after CRS. Here we have shown that SAPESUCA is able to quantify the actual intraperitoneal chemotherapeutic dose based on PSA after CRS. Our intended prospective monocentric phase I SAPESUCA-HIPEC study investigates if intraperitoneal chemotherapeutic dose adjustment during HIPEC calculated on actual PSA and not BSA by SAPESUCA reduces postoperative morbidity with beneficial effects on long-term survival in colorectal cancer patients with peritoneal metastases.

## 5. Conclusions

Here we present SAPESUCA, the first free web-based application enabling standardized quantification and thus comparability of the resected and remaining PSA after CRS. SAPESUCA is a valuable tool to improve the lacking inter- and intra-individual and inter-institutional comparability of patients with peritoneal diseases treated with CRS and HIPEC. Furthermore, our Shiny app may provide a powerful tool for calculating the intraperitoneal chemotherapeutic dose during HIPEC. The app’s user-friendly interface, free accessibility, data visualization options, and robust analytical capabilities make it an important resource for researchers and cytoreductive surgeons.

Multicentric application and ongoing research of SAPESUCA are necessary for further validation.

## Figures and Tables

**Table 1:** Patients’ baseline characteristics and treatment-related parameters (Completeness of Cytoreduction [CC] Score).

Parameter			N = 27 (100%)
Median age [years] (range)		56 (22–75)	
Median BMI [kg/m^2^] (range)		25.7 (15.2–31.2)	
Sex	Male		16 (59%)
Tumor histology	Adenocarcinoma Mucinous Signet ring cell		12 (44%) 12 (44%) 3 (11%)
Tumor localization	Right colon Transverse colon Left colon Sigmoid colon		8 (30%) 5 (18%) 4 (15%) 10 (37%)
Previous abdominal surgery for colon cancer	Yes		18 (67%)
Previous chemotherapy	Yes		18 (67%)
Median PCI (range)		9 (1–21)	
Median operative time [min] (range)		352 (190–632)	
CC-score	0 1		26 (96%) 1 (4%)
Cytoreduction	Greater omentum Liver metastasis Gallbladder Lesser omentum Stomach Spleen Pancreas Small bowel Colon Rectum Bladder Uterus Ovaries Other organs		20/27 (74%) 5/27 (19%) 7/27 (26%) 11/27 (41%) 0/27 (0%) 5/27 (19%) 2/27 (7%) 15/27 (56%) 21/27 (78%) 12/27 (44%) 1/27 (4%) 8/27 (30%) 9/27 (33%) 9/27 (33%)
Stoma creation	Yes		14 (52%)
30-day complication	Yes		10 (37%)
Clavien-Dindo complication rate	0 I II IIIa IIIb		17 (63%) 2 (7%) 2 (7%) 2 (7%) 4 (15%)
Median length of hospital stay (days) (range)	14 (7–61)		
Median length of ICU stay (days) (range)	2 (1–9)		

## Data Availability

Data supporting results are harbored by an in-hospital database. Regulatory issues do not allow the provision of a link to analyzed data sets.

## Supplementary Materials

The following supporting information can be downloaded at: https://www.mdpi.com/article/10.3390/cancers15123134/s1; Table S1. Anatomical peritoneal regions; Table S2. Total peritoneal surface area before and after cytoreductive surgery; Table S3. Resected peritoneal surface area; The software code is stored on a package available on GitHub.

## Abbreviations

SAPESUCA	SAlzburg PEritoneal SUrface CAlculator
HIPEC	Hyperthermic Intraperitoneal Chemotherapy
CRS	Cytoreductive Surgery
PSA	Peritoneal Surface Area
PCI	Peritoneal Cancer Index
SPCI	Simplified Peritoneal Cancer Index
CC	Completeness of Cytoreduction
PSS	Prior Surgical Score
PESUCA	PEritoneal SUrface CAlculator
BSA	Body Surface Area
KPI	Key Performance Indicator
GUI	Graphical User Interface
CT	Computed Tomography
