# Supplementary material for: The SAlzburg PEritoneal SUrface CAlculator (SAPESUCA): The First Web-Based Application for Peritoneal Surface Area Quantification
Source: Cancers (Basel). 2023 Jun 10;15(12):3134. doi: 10.3390/cancers15123134 (PMC10296656; doi:10.3390/cancers15123134)
Supplement: Supplementary file 1 [file cancers-15-03134-s001.zip › cancers-2256074-supplementary.pdf]

Supplementary Materials:

Table S1. Forty anatomical peritoneal regions grouped in supra-, and infracolic visceral, and parietal peritoneum based on [1] and their rearrangement into 13 Peritoneal Cancer Index (PCI) regions shown in *(brackets)* based on [2].

No.	Supracolic peritoneum, visceral area	Supracolic peritoneum, parietal area	Infracolic peritoneum, visceral area	Infracolic peritoneum, parietal area
**1**	Liver *(1: Right upper)*	Right diaphragmatic wall *(1: Right upper)*	Mesentery *(9-12: Upper/Lower jejunum, Upper/Lower ileum)*	Right antero-lateral infraumbilical wall *(0: Central)*
**2**	Gastrocolic ligament *(0: Central)*	Left diaphragmatic wall *(3: Left upper)*	Jejunum-ileum *(9-12: Upper/Lower jejunum, Upper/Lower Ileum)*	Left antero-lateral infraumbilical wall *(0: Central)*
**3**	Stomach *(3: Left upper)*	Right antero-lateral supraumbilical wall *(0: Central)*	Greater omentum *(0: Central)*	Left dorsal infracolic parietal wall *(4: Left flank)*
**4**	Spleen *(3: Left upper)*	Left antero-lateral supraumbilical wall *(0: Central)*	Sigmoid colon *(5: Left lower)*	Right dorsal infracolic parietal wall *(8: Right flank)*
**5**	Transverse mesocolon: superior layer *(0: Central)*	Right dorsal supracolic parietal wall *(1: Right upper)*	Transverse colon *(0: Central)*	Left lateral pelvic wall *(5: Left lower)*
**6**	Lesser omentum *(2: Epigastrium)*	Left dorsal supracolic parietal wall *(3: Left upper)*	Transverse mesocolon: inferior layer *(0: Central)*	Right lateral pelvic wall *(7: Right lower)*
**7**	Falciform ligament *(2: Epigastrium)*		Caecum v. appendix ascending colon *(8: Right flank)*	
**8**	Pancreas *(0: Central)*		Sigmoid mesocolon *(5: Left lower)*	
**9**	Gastrosplenic ligament *(3: Left upper)*		Uterus and broad ligaments *(6: Pelvis)*	
**10**	Teres ligament *(2: Epigastrium)*		Rectum *(6: Pelvis)*	
**11**	Duodenum *(1: Right upper)*		Descending colon *(4: Left flank)*	
**12**	Left triangular ligament *(2: Epigastrium)*		Urinary bladder *(6: Pelvis)*	
**13**	Gall bladder *(1: Right upper)*			
**14**	Lienorenal ligament *(3: Left upper)*			
**15**	Right triangular ligament *(1: Right upper)*			
**16**	Abdominal esophagus			

	(3: Left upper)

Table S2. Peritoneal surface area (cm²) before and after cytoreductive surgery in all 13 Peritoneal Cancer Index (PCI) regions. Data of all procedures are shown as mean ± SD and range (minimum [min] and maximum [max]).

Region	Peritoneal surface area before cytoreductive surgery				Peritoneal surface area after cytoreductive surgery			
	mean	*SD*	*min*	*max*	*mean*	*SD*	*min*	*max*
**Central**	3,163	332	2,386	3,163	1,646	755	62	1,646
**Right Upper**	2,219	232	1,673	2,219	1,995	440	928	1,995
**Epigastrium**	939	99	708	939	447	185	0	447
**Left Upper**	1,723	181	1,299	1,723	1,562	341	474	1,562
**Left Flank**	549	57	414	549	288	240	4	288
**Left Lower**	1,128	118	851	1,128	292	487	0	292
**Pelvis**	605	64	456	605	345	193	5	345
**Right Lower**	337	35	254	337	118	129	0	118
**Right Flank**	650	68	490	650	305	300	0	305
**Upper Jejunum**	1,825	191	1,376	1,825	1,812	179	1,376	1,812
**Lower Jejunum**	1,825	191	1,376	1,825	1,804	171	1,376	1,804
**Upper Ileum**	1,825	191	1,376	1,825	1,667	515	0	1,667
**Lower Ileum**	1,825	191	1,376	1,825	1,399	646	0	1,399

Table S3. Resected peritoneal surface area in cm² in all 13 Peritoneal Cancer Index (PCI) regions. Data of all patients are shown as mean ± SD and range (minimum [min] and maximum [max]).

Region	mean	SD	min	max
**Central**	1,517	737	691	1,517
**Right Upper**	223	351	0	223
**Epigastrium**	492	209	0	492
**Left Upper**	161	292	0	161
**Left Flank**	260	244	0	260
**Left Lower**	836	512	0	836
**Pelvis**	259	187	0	259
**Right Lower**	218	141	0	218
**Right Flank**	345	306	0	345
**Upper Jejunum**	13	32	0	13
**Lower Jejunum**	21	58	0	21
**Upper Ileum**	158	507	0	158
**Lower Ileum**	425	649	0	425

References

1. Albanese, A.M.; Albanese, E.F.; Mino, J.H.; Gomez, E.; Gomez, M.; Zandomeni, M.; and Merlo, A.B. Peritoneal surface area: measurements of 40 structures covered by peritoneum: correlation between total peritoneal surface area and the surface calculated by formulas. *Surg Radiol Anat* **2009**, *31*(5), p. 369-377.
2. Jacquet, P. and Sugarbaker, P.H. Clinical research methodologies in diagnosis and staging of patients with peritoneal carcinomatosis. *Cancer Treat Res* **1996**, *82*, p. 359-374.
